# Self-reported oral health among Brazilian adults: results from the National Health Surveys 2013 and 2019

**DOI:** 10.1590/SS2237-9622202200014.especial

**Published:** 2022-08-01

**Authors:** Rafael Bello Corassa, Carlos José de Paula Silva, Janice Simpson de Paula, Érika Carvalho de Aquino, Luciana Monteiro Vasconcelos Sardinha, Paula Aryane Brito Alves

**Affiliations:** 1 Ministério da Saúde, Secretaria de Vigilância em Saúde, Brasília, DF, Brazil; 2 Universidade Federal de Minas Gerais, Faculdade de Odontologia, Belo Horizonte, MG, Brazil; 3 Universidade Federal dos Vales do Jequitinhonha e Mucuri, Faculdade de Ciências Biológicas e da Saúde, Diamantina, MG, Brazil

**Keywords:** Oral Health, Health Surveys, Public Health Surveillance, Health Status Disparities, Cross-Sectional Studies

## Abstract

**Objective:**

To evaluate indicators of oral health conditions and behaviours among Brazilian adults in the 2019 National Health Survey (PNS) and analyse the evolution of those indicators compared to the 2013 PNS.

**Methods:**

Cross-sectional study. Prevalence ratios of oral health conditions and behaviours, in 2019, were estimated by demographic characteristics. Risk ratios were computed using Poisson regression, and absolute differences (Dif.) between indicators in 2013 and 2019 were calculated.

**Results:**

Prevalence of brushing teeth twice a day, using toothbrush/toothpaste/floss and edentulism were, respectively, 93.6% (95%CI 93.3;93.9), 63.0% (95%CI 62.3;63.6) and 10.3% (95%CI 9.93;10.7). There was increase in prevalence of brushing teeth ≥ 2 a day (Dif. = 4.5; 95%CI 3.9;5.1), using toothbrush/toothpaste/floss (Dif. = 10.0; 95%CI 8.6;11.3) and a decrease in prevalence of edentulism (Dif. = -0.7; 95%CI -1.3;-0.1).

**Conclusion:**

Respondents who were younger, more educated, with higher income and lived in urban areas had better oral health indicators. Most indicators demonstrated positive improvement.

**Study contributions**

**Main results**

There was improvement in almost all oral health indicators in the period, especially those related to oral hygiene. Inequalities were observed in the distribution and evolution of the indicators analyzed, with worse results among the individuals most vulnerable.

**Implications for services**

Despite the improvement in oral health conditions, the inequalities in the indicators analyzed reinforce the need to strengthen oral health care in the Brazilian National Health System (SUS), ensuring equitable access to oral health in Brazil.

**Perspectives**

The inequalities in the analyzed indicators reinforce the importance of SUS for the democratization of access to oral health in Brazil. The limitations of self-reported measures, however, reinforce the importance of conducting clinical examinations in conjunction with surveys.

## Introduction

In the past decades, there has been considerable improvement in the oral health conditions of the Brazilian population.^1^ However, this process of improvement has been marked by socioeconomic and cultural inequalities, as well as difficulties in structuring oral health care according to the principles of equity, universality and comprehensive health care.^2^ Despite the improvement in indicators, oral diseases are still an important public health problem, due to their high prevalence in the Brazilian population.^1^

Population surveys are important strategies in terms of assessing the magnitude of oral health problems in the population, of identifying the collective health profile and collective health needs, and also in defining and monitoring health indicators, being essential for the formulation of public policies and their evaluation.^3^

National surveys that address the oral health condition of the Brazilian population are scarce, despite the relevance of the topic. Noteworthy are the epidemiological studies conducted by the Ministry of Health in 1986 and 1996, the National Oral Health Survey (*Pesquisa Nacional de Saúde Bucal* - SB Brasil) of 2003 and 2010,^4^ and the 2013 National Health Survey (PNS).^1^

Comparison of the results of these surveys showed that, over the years, the oral health conditions of the Brazilian population have improved. The index of decayed, missing and filled teeth (DMFT) is an indicator used by the World Health Organization (WHO) to verify the severity of dental caries in the population. In 1986, the DMFT in 12-year-old children was 6.7 teeth, a value considered very high worldwide.^5^ ^,^ ^6^ In 2000 this index dropped to 2.8 and, in 2010, to 2.1.^5^ In adults aged 35 to 44 years, the average DMFT reached 22.5 teeth in 1986,^6^ dropping to 20.1 in 2000, and to 16.3 in 2010.^5^ Such improvement is attributed to factors such as the fluoridation of supply waters, the incorporation of fluoride into toothpastes, the expansion of access to preventive practices, the improvement of human development indicators and the implementation of the National Oral Health Policy (*Política Nacional de Saúde Bucal*) in Brazil.^4^

The implementation of a National Oral Health Policy, in 2004, through the program Brasil Sorridente, represented a milestone for oral health care within the scope of public health in Brazil. This policy contributed to the expansion and training of Oral Health Teams within the scope of the Family Health Strategy, to the creation of Dental Specialty Centers and Regional Dental Prosthesis Laboratories, and to the development of surveillance and oral health education and promotion actions, revealing its fundamental role in expanding access to care and in improving the indicators in the country.^5^

Nevertheless, the continuous assessment of oral health indicators is essential to understand and identify collective needs, as well as to provide data and information that can support the formulation of public policies and guide the organization of health services.^3^

In light of the above, the present study aimed to analyze indicators of conditions and behaviors related to the oral health of Brazilian adults in the 2019 National Health Survey (PNS), and its evolution in relation to 2013.

## Methods

A cross-sectional study was carried out, using data from the 2013 and 2019 editions of the PNS.

The PNS is a population-based national household survey representative of the population residing in private households in Brazil. The 2013 PNS used a representative sample of the adult population aged ≥ 18 years, while the 2019 PNS used a representative sample of the population aged ≥ 15 years. For the selection of participants, a complex sampling process was carried out by clusters, in three stages: (i) stratification of the Primary Sampling Units (PSU), composed of one or more census sectors, and random selection with probability of selection proportional to the number of permanent private households; (ii) selection of households in each PSU, based on the most recent update available from the National Registry of Addresses for Statistical Purposes (CNEFE), by simple random sampling; (iii) simple random selection of an eligible resident, based on the list of residents prepared at the time of the interview.

The sample size of the 2013 PNS was scaled based on expected estimates of indicators and desired coefficients of variation. Details on the sampling plan and weighting of the 2013 PNS were published by Damacena et al.^7^ and Souza-Júnior et al.^8^ The sample size of the 2019 PNS was calculated based on indicators from the 2013 edition. More details on the sampling plan, data collection and weighting process were published by Stopa et al.^10^ and by the Brazilian Institute of Geography and Statistics (IBGE).^9^

Considering the differences in the age groups of the samples of the 2013 and 2019 editions, respondents under 18 years of age were excluded from this study, in order to ensure the comparability of the estimates. Data on oral health were obtained from the “Module U” of the questionnaire, and six indicators were selected, characterized as binary variables (yes/no), referring to self-reported oral hygiene practices and oral health conditions, constructed on the basis of items available in the questionnaire:

- Brushing teeth at least twice a day: *How often do you use a toothbrush for oral hygiene?* (three times or more a day; twice a day; once a day; I do not brush my teeth everyday);
- Use of toothbrush, toothpaste and dental floss to clean teeth: *What do you use to clean your mouth?* [*Toothbrush?* (yes; no); *Toothpaste?* (yes; no); *Dental floss?* (yes; no)], considering the individuals who responded affirmatively to the three questions;
- Rates his/her oral health as good or very good: *In general, how do you rate your oral health (teeth and gums)?* (very good; good; fair; bad; very bad);
- Lost all teeth: *Thinking back on your upper permanent teeth, have you lost any?* (no/yes, how many; yes, I lost all my upper teeth), and *Thinking back on your lower permanent teeth, have you lost any?* (no/yes, how many; yes, I lost all my lower teeth), considering the individuals who reported having lost all their teeth in the upper and lower arches, or who reported having lost 16 permanent teeth in both upper and lower arches;
- Use of any type of dental prosthesis, among those who had tooth loss: *Do you use any type of dental prosthesis (artificial tooth, implant, denture)?* (yes; no);
- Intense or very intense difficulty in eating due to teeth or prosthesis problems: *How difficult is it for you to eat because of problems with your teeth or denture?* (none; mild; regular; intense; very intense).

The demographic variables analyzed were: sex (male; female), age group (18-29; 30-39; 40-59; ≥ 60 years), education (no schooling and incomplete elementary education; complete elementary education and incomplete high school; complete high school and incomplete higher education; complete higher education), race/skin color (White; Black; Brown), household income per capita in minimum wages (MWs), (up to 1, more than 1 to 3, more than 3), place of residence (urban; rural). Regarding race/skin color, separate results were not presented for oral health indicators among Indigenous and Yellow race/skin color due to the lack of representativeness for these population strata in the research.

The prevalence of each indicator and their respective 95% confidence intervals (95%CI) were calculated according to demographic characteristics. Bivariate analyzes were performed using the chi-squared test and prevalence ratios (PR), and their 95%CI, were calculated using Poisson regression. Comparisons between the prevalence of each indicator in the 2013 and 2019 editions of the PNS were performed using the *nlcom* command, a post-estimation technique that enables conducting non-linear combinations of estimators, and whose results are presented as absolute difference between the prevalence (Dif.) and 95%CI. The difference between the prevalence in the two periods was tested by calculating the z statistics . The analyzes were performed considering a significance level of 5%.

Considering the complex sample design of the research, the analyzes were performed considering the strata, primary sampling units and sample weights, using the survey module (svy) of the Stata program, version 14.2 (StataCorp. 2015. Stata Statistical Software: Release 14. College Station, TX: StataCorp LP).

Data referring to the two editions of the PNS are publicly available on the website of the IBGE, and were extracted from the website on April 10, 2021. Both editions of the PNS were approved by the National Committee for Ethics in Research of the National Health Council (PNS 2013: opinion No. 328,159; PNS 2019: opinion No. 3,529,376).

## Results

In the 2019 PNS, a total of 90,846 individuals were interviewed. After excluding those under 18 years of age, a total sample of 88,531 adults, domiciled in Brazil, was analyzed. The 2013 edition presented a total of 60,202 adult respondents, domiciled in Brazil. 

In 2019, 93.6% (95%CI 93.3;93.9) of the sample reported brushing their teeth at least twice a day (Table 1). Higher prevalence was observed among people with complete higher education (PR = 1.12; 95%CI 1.11;1.13), household income per capita greater than 3 MWs (PR = 1.07; 95%CI 1.06;1.07) and residents of urban areas (PR = 1.08; 95%CI 1.07;1.09) (Table 2). The prevalence of toothbrush, toothpaste and dental floss use in oral hygiene was 63.0% (95%CI 62.2;63.7). Higher prevalence was observed among females (PR = 1.17; 95%CI1.15;1.20), people with complete higher education (PR = 2.30; 95%CI 2.24;2.37), with an income above 3 MWs (PR = 1.57; 95%CI 1.53;1.66) and living in urban areas (PR = 1.55; 95%CI 1.51;1.60).

**Table 1:** Description of self-reported hygiene practices and oral health among adults aged ≥ 18 years in the 2019 (n = 88,531) and 2013 (n = 60,202) editions of the National Health Survey

Variables	2019		2013		Difference^a^		p-value^d^
	%	95%CI^c^	%	95%CI^c^	Dif.	95%CI^c^	
Brushing teeth at least twice a day	93.6	93.3;93.9	89.1	88.6;89.6	4.5	3.9;5.1	< 0.001
Using a toothbrush, toothpaste and dental floss for teeth cleaning	63.0	62.2;63.7	53.0	52.0;54.0	10,0	8.6;11.3	< 0.001
Self-assessment of oral health as good or very good	69.7	69.1;70.3	67.4	66.7;68.2	2.2	1.2;3.3	< 0.001
Total tooth loss	10.3	9.9;10.7	11.0	10.5;11.5	-0.7	-1.3;-0.1	0.021
Use of some type of dental prosthesis^b^	45.9	45.2;46.6	46.5	45.6;47.4	-0.6	-1.8;0.6	0.339
Intense or very intense difficulty in eating due to teeth or prosthesis problems	1.8	1.7;2.0	1.5	1.4;1.7	0.3	0.1;0.6	0.017

a) Dif.: Absolute difference between the 2019 and 2013 prevalences; b) Only individuals who reported at least one tooth loss were considered; c) 95%CI: 95% Confidence interval; d) P-values obtained by calculating the z-statistics using nonlinear combinations of estimators by means of the *nlcom* command.

**Table 2:** Prevalence of brushing teeth ≥ 2 a day and use of toothbrush, toothpaste and dental floss for oral hygiene among adults, according to demographic variables, Brazil, 2019 National Health Survey (n = 88,531)

	Brushing teeth at least twice a day							Use of toothbrush, toothpaste and dental floss for teeth cleaning					
	n^a^	%	95%CI^c^	PR^d^	95%CI^c^	p-value^e^		n^a^	%	95%CI^c^	PR^d^	95%CI^c^	p-value^e^
**Sex**													
Male	37,402	91.7	91.2;92.2	1.00		< 0.001		21,715	57.6	56.7;58.6	1.00		< 0.001
Female	44,279	95.3	95.0;95.6	1.04	1.03;1.05			29,134	67.7	66.9;68.4	1.17	1.15;1.20	
**Age group (years)**													
18 to 29	14,836	96,0	95.4;96.7	1.00		< 0.001		10,211	69.8	68.5;71.1	1.00		< 0.001
30 to 39	17,493	96.7	96.3;97.2	1.01	1.00;1.02			12,772	74.8	73.7;75.9	1.07	1.05;1.10	
40 to 59	30,238	94.6	94.2;95.0	0.99	0.98;0.99			19,529	65.8	64.8;66.8	0.94	0.92;0.96	
≥ 60	19,114	86.5	85.8;87.2	0.90	0.89;0.91			8,337	39.8	38.6;41.0	0.57	0.55;0.59	
**Education**													
No schooling and incomplete elementary education	30,377	87.5	86.9;88.0	1.00		< 0.001		11,903	38.5	37.5;39.4	1.00		< 0.001
Complete elementary education and incomplete high school	11,318	94.4	93.6;95.2	1.08	1.07;1.09			6,857	61,0	59.5;62.6	1.59	1.53;1.65	
Complete high school and incomplete higher education	26,594	97.3	97.0;97.7	1.11	1.10;1.12			20,181	76.5	75.6;77.4	1.99	1.94;2.04	
Complete higher education	13,392	98.2	97.9;98.6	1.12	1.11;1.13			11,908	88.6	87.6;89.5	2.30	2.24;2.37	
**Race/skin color^b^**													
White	30,273	94.5	94.1;94.9	1.00		< 0.001		20,753	68.1	67.1;69.2	1.00		< 0.001
Black	9,239	92.7	91.8;93.5	0.98	0.97;0.99			5,519	59.1	57.4;60.7	0.87	0.84;0.89	
Brown	40,927	93,0	92.6;93.4	0.98	0.98;0.99			23,767	58.7	57.8;59.6	0.86	0.84;0.88	
**Household income *per capita* (minimum wage)**													
Up to 1	43,555	91.4	90.9;91.8	1.00		< 0.001		22,963	53.4	52.5;54.3	1.00		< 0.001
More than 1 to 3	27,874	95.5	95.1;95.8	1.04	1.04;1.05			19,206	69.6	68.6;70.6	1.30	1.28;1.33	
More than 3	10,230	97.5	96.9;97.9	1.07	1.06;1.07			8,660	83.8	82.5;85.0	1.57	1.53;1.66	
**Place of residence**													
Urban	64,237	94.6	94.3;94.9	1.08	1.07;1.09	< 0.001		43,203	66.2	65.5;67.0	1.55	1.51;1.60	< 0.001
Rural	17,444	87.7	86.8;88.6	1.00				7,646	42.6	41.3;43.9	1.00		

a) Unweighted values; b) Indigenous and Yellow race/skin color represented 1.46% of the sample and were not shown due to lack of representativeness of such groups in the National Health Survey (PNS); c) 95%CI: 95% Confidence interval; d) PR: Prevalence ratio; e) P-value: Chi-squared test.

Regarding oral health self-perception, 69.7% (95%CI 69.1;70.3) of the sample rated their oral health as good or very good (Table 1). Higher prevalence was observed among people with complete higher education (PR = 1.39; 95%CI 1.36;1.41), with an income over 3 MWs (PR = 1.33; 95%CI 1.31;1.46) and residing in urban areas (PR = 1.14; 95%CI 1.11;1.16) (Table 3). The prevalence of self-reported edentulism was 10.3% (95%CI 9.9;10.7) and the highest prevalences were observed among females (PR = 1.62; 95%CI 1.51;1.73), the elderly (PR = 211.69; 95%CI 122.34;366.29) and lower prevalence among people with higher education (PR = 0.08; 95%CI 0.07;0.10) and an income above 3 MWs (PR = 0.44; 95%CI 0.38;0.50) (Table 3).

**Table 3:** Prevalence of self-rated health as good/very good and edentulism among domiciled adults, according to selected demographic variables, Brazil, National Health Survey, 2019 (n = 88,531)

	Self-rated oral health as good or very good							Loss of all teeth					
	n^a^	%	95%CI^c^	PR^d^	95%CI^c^	p-value^e^		n^a^	%	95%CI^c^	PR^d^	95%CI^c^	p-value^e^
**Sex**													
Male	27,462	68.3	67.4;69.1	1.00		< 0.001		3,984	7.7	7.3;8.2	1.00		< 0.001
Female	32,423	70.9	70.1;71.7	1.04	1.02;1.06			6,714	12.5	12.0;13.0	1.62	1.51;1.73	
**Age group (years)**													
18 to 29	11,224	74.9	73.7;76.1	1.00		< 0.001		26	0.2	0.1;0.3	1.00		< 0.001
30 to 39	12,923	72.5	71.4;73.7	0.97	0.95;0.99			80	0.4	0.3;0.5	2.10	1.10;4.01	
40 to 59	21,015	66.7	65.6;67.7	0.89	0.87;0.91			2,234	6.3	5.8;6.8	36.04	20.71;62.73	
≥ 60	14,723	66.5	65.4;67.6	0.89	0.87;0.91			8,358	36.8	35.7;37.9	211.69	122.34;366.29	
**Education**													
No schooling and incomplete elementary education	21,188	60.4	59.4;61.3	1.00		< 0.001		8,606	23.5	22.7;24.4	1.00		< 0.001
Complete elementary education and incomplete high school	7,777	65.8	64.2;67.3	1.09	1.06;1.12			856	5.9	5.3;6.7	0.25	0.22;0.28	
Complete high school and incomplete higher education	19,669	74.3	73.4;75.1	1.23	1.21;1.25			922	2.7	2.4;3.0	0.11	0.10;0.13	
Complete higher education	11,251	83.6	82.5;84.7	1.39	1.36;1.41			314	2.0	1.6;2.4	0.08	0.07;0.10	
**Race/skin color^b^**													
White	23,588	74.8	73.9;75.7	1.00		< 0.001		4,144	10.9	10.3;11.5	1.00		< 0.001
Black	6,325	63.2	61.6;64.8	0.84	0.82;0.87			1,153	9.5	8.6;10.4	0.87	0.78;0.97	
Brown	29,104	66.3	65.5;67.2	0.89	0.87;0.90			5,249	9.9	9.5;10.4	0.91	0.85;0.98	
**Household income *per capita* (minimum wage)**													
Up to 1	29,778	62.9	62.1;63.7	1.00		< 0.001		6,206	11.2	10.8;11.7	1.00		< 0.001
More than 1 to 3	21,433	74.6	73.6;75.5	1.18	1.16;1.21			3,856	10.7	10.1;11.3	0.95	0.89;1.02	
More than 3	8,655	83.9	82.6;85.1	1.33	1.31;1.36			636	4.9	4.3;5.5	0.44	0.38;0.50	
**Place of residence**													
Urban	47,363	70.9	70.2;71.5	1.14	1.11;1.16	< 0.001		7,443	9.6	9.3;10.0	0.67	0.63;0.72	< 0.001
Rural	12,522	62.3	61.1;63.5	1.00				3,255	14.3	13.5;15.1	1.00		

a) Unweighted values; b) Indigenous and Yellow race/skin color represented 1.46% of the sample and were not shown due to lack of representativeness of such groups in the National Health Survey (PNS); c) 95%CI: 95% Confidence interval; d) PR: Prevalence ratio; e) P-value: Chi-squared test.

The proportion of people aged ≥ 18 years who reported using dental prostheses, among those who reported tooth loss, was 45.9% (95%CI 45.2;46.6). Higher prevalence was found among females (PR = 1.22; 95%CI 1.18;1.26), the elderly (PR = 11.90; 95%CI 9.98;14.20) and people with an income between 1 and 3 MWs (PR = 1.29; 95%CI 1.35;1.33) and greater than 3 MWs (PR = 1.35; 95%CI 1.29;1.41) (Table 4). The prevalence of individuals who reported intense or very intense difficulty in eating due to problems with their teeth or dentures was 1.8% (95%CI 1.7;2.0), being higher among females (PR = 1 .75; 95%CI 1.46;2.10), those aged between 40 and 59 years (PR = 3.87; 95%CI 2.63;5.71), the elderly (PR = 6.43; 95%CI 4.47;9.25) and individuals of black race/skin color (RP = 2.14 95%CI 1.58;2.90) (Table 4).

**Table 4:** Prevalence of prosthesis use and eating difficulties due to prosthesis or teeth problems among domiciled adults, according to selected demographic variables, Brazil, 2019 National Health Survey (n = 88,531)

	Use of some type of dental prosthesis^a^							Intense or very intense difficulty in eating due to teeth or prosthesis problems					
	n^b^	%	95%CI^d^	PR^e^	95%CI^d^	p-value^f^		n^b^	%	95%CI^d^	PR^e^	95%CI^d^	p-value^f^
**Sex**													
Male	13,066	41.0	39.9;42.0	1.00		0.001		750	1.3	1.2;1.5	1.00		< 0.001
Female	19,335	50.0	49.0;50.9	1.22	1.18;1.26			1,173	2.3	2.0;2.6	1.75	1.46;2.10	
**Age group (years)**													
18 to 29	429	6.3	5.3;7.5	1.00		< 0.001		104	0.6	0.4;0.8	1.00		< 0.001
30 to 39	2,096	17.5	16.3;18.7	2.77	2.29;3.34			186	0.8	0.7;1.0	1.49	0.99;2.23	
40 to 59	13,834	49.5	48.4;50.6	7.85	6.58;9.37			762	2.2	1.8;2.6	3.87	2.63;5.71	
≥ 60	16,042	75.0	74.1;76.0	11.90	9.98;14.20			871	3.6	3.2;4.1	6.43	4.47;9.25	
**Education**													
No schooling and incomplete elementary education	18,010	56.4	55.5;57.4	1.00		< 0.001		1,380	3.8	3.4;4.2	1.00		< 0.001
Complete elementary education and incomplete high school	3,796	40.6	38.7;42.5	0.72	0.69;0.75			222	1.5	1.2;1.8	0.39	0.31;0.49	
Complete high school and incomplete higher education	6,774	34.5	33.2;35.7	0.61	0.59;0.64			254	0.7	0.6;0.9	0.18	0.15;0.23	
Complete higher education	3,821	42.9	41.2;44.7	0.76	0.73;0.80			67	0.5	0.3;0.7	0.12	0.08;0.20	
**Race/skin color^c^**													
White	13,077	50.7	49.6;51.9	1.00		< 0.001		579	1.5	1.2;1.9	1.00		< 0.001
Black	3,271	40.7	38.8;42.7	0.80	0.76;0.85			293	3.2	2.6;4.0	2.14	1.58;2.90	
Brown	15,544	42.5	41.5;43.5	0.84	0.81;0.87			1,024	1.9	1.7;2.1	1.23	0.98;1.54	
**Household income *per capita* (minimum wage)**													
Up to 1	15,959	40.2	39.3;41.0	1.00		< 0.001		1,365	2.6	2.3;2.9	1.00		< 0.001
More than 1 to 3	12,238	51.7	50.5;52.9	1.29	1.25;1.33			475	1.3	1.1;1.5	0.51	0.41;0.62	
More than 3	4,199	54.2	52.0;56.3	1.35	1.29;1.41			83	0.6	0.4;0.8	0.22	0.15;0.33	
**Place of residence**													
Urban	24,847	46.4	45.6;47.2	1.07	1.03;1.11	< 0.001		1,352	1.7	1.6;2.0	0.69	0.58;0.81	< 0.001
Rural	7,554	43.4	42.0;44.7	1.00				571	2.5	2.2;2.9	1.00		

a) Only individuals who reported the loss of at least one tooth were considered; b) Unweighted values; c) Indigenous and yellow race/skin color represented 1.46% of the sample and were not shown due to lack of representativeness of such groups in the National Health Survey (PNS); d) 95%CI: 95% Confidence interval; e) PR: Prevalence ratio; f) P-value: Chi-squared test.

Between 2013 and 2019, improvements were observed in almost all indicators evaluated, except for the use of dental prosthesis among those who had tooth loss. There was an increase in the prevalence of using toothbrush, toothpaste and dental floss for oral hygiene (Dif. = 10.0; 95%CI 8.6;11.3) and brushing at least twice a day (Dif. = 4.5; 95%CI 3.9;5.1).

Table 5 shows the differences between the prevalence of indicators, according to demographic variables, in the 2013 and 2019 editions of the PNS. There was an increase in the prevalence of brushing at least twice a day among the elderly (Dif. = 13.1; 95%CI 11.3;14.8) and among people with no schooling or with incomplete elementary education (Dif. = 7.8; 95%CI 6.7;8.9). Regarding the use of toothbrush, toothpaste and dental floss, there was an increase in prevalence among individuals of black race/skin color (Dif. = 15.5; 95%CI 12.4;18.6) and residing in urban areas. (Dif. = 12.2; 95%CI 9.9;14.6). There was also a reduction in the prevalence of edentulism among the elderly (Dif. = -4.7; 95%CI -6.8;-2.6) and adults aged 40 to 59 years (Dif. = -3.6; 95%CI -4.5;-2.6). On the other hand, a reduction in the prevalence of the use of prosthesis in adults with tooth loss was identified, reaching less than 8 percentage points in the 30 to 39 age group (95%CI -10.0;-6.0).

**Table 5:** Absolute differences between the prevalence of hygiene practices and oral health conditions among domiciled adults, according to selected demographic variables, Brazil, 2019 (n = 88,531) and 2013 (n = 60,202) National Health Survey

	Brushing teeth at least twice a day		Use of toothbrush, toothpaste, and dental floss for teeth cleaning		Self-rated oral health as good or very good		Loss of all teeth		Use of some kind of dental prosthesis^a^		Intense or very intense difficulty eating due to problems with teeth or prosthesis	
	Dif.^b^	95%CI^g^	Dif.^b^	95%CI^g^	Dif.^b^	95%CI^g^	Dif.^b^	95%CI^g^	Dif.^b^	95%CI^g^	Dif.^b^	95%CI^g^
**Sex**												
Male	3.8^f^	3.1;4.5	10.6^f^	9.1;12.0	2.0^e^	0.7;3.3	-0.8	-1.7;0.0	-1.4	-2.9;0.1	0.6^e^	0.2;1.0
Female	5.2^f^	4.3;6.2	9.2^f^	7.5;11.0	2.5^e^	1.1;3.9	-0.6	-1.4;0.2	0.2	-1.5;2.0	-0.1	-0.4;0.2
**Age group (years)**												
18 to 29	1.1^d^	0.1;2.0	8.4^f^	6.2;10.6	0.9	-0.9;2.7	0.1^e^	0.0;0.2	-1.5	-3.2;0.1	0.0	-0.3;0.3
30 to 39	2.1^f^	1.4;2.9	9.9^f^	7.9;11.9	2.4^e^	0.6;4.2	-0.2	-0.4;0.0	-8.0^f^	-10.0;-6.0	0.1	-0.2;0.4
40 to 59	5.1^f^	4.2;6.0	14.1^f^	12.3;15.9	3.2^f^	1.5;4.8	-3.6^f^	-4.5;-2.6	-6.3^f^	-8.1;-4.5	0.3	-0.2;0.8
≥ 60	13.1^f^	11.3;14.8	10.7^f^	8.4;13.0	4.2^f^	2.2;6.3	-4.7^f^	-6.8;-2.6	2.9^e^	1.0;4.7	0.3	-0.4;1.0
**Education**												
No schooling and incomplete elementary education	7.8^f^	6.7;8.9	9.3^f^	7.7;10.8	2.9^f^	1.4;4.4	0.7	-0.6;2.0	1.8^d^	0.1;3.5	0.6	0.0;1.1
Complete elementary education and incomplete high school	2.1^e^	0.8;3.5	8.5^f^	6.0;11.0	-1.0	-3.3;1.2	-0.6	-1.8;0.7	-1.2	-4.2;1.8	0.7^f^	0.3;1.1
Complete high school and incomplete higher education	1.8^f^	1.1;2.5	6.8^f^	5.2;8.4	1.0	-0.5;2.4	0.1	-0.3;0.6	-0.7	-2.7;1.3	0.3^d^	0.0;0.5
Complete higher education	0.5	-0.2;1.2	5.4^f^	3.6;7.1	0.2	-1.7;2.0	-0.1	-0.7;0.5	-3.1	-6.5;0.3	0.3^d^	0.1;0.5
**Race/skin color^c^**												
White	4.2^f^	3.4;5.0	8.2^f^	6.5;10.0	1.7^d^	0.3;3.0	-0.7	-1.6;0.2	-2.1^d^	-3.9;-0.3	0.2	-0.2;0.6
Black	4.6^f^	2.9;6.4	15.5^f^	12.4;18.6	2.0	-0.9;4.9	-1.5	-3.4;0.4	0.0	-3.4;3.4	1.0^d^	0.0;2.0
Brown	5.1^f^	4.2;5.9	11.6^f^	9.9;13.3	3.9^f^	2.5;5.3	-0.5	-1.3;0.4	1.4	-0.3;3.0	0.2	-0.1;0.5
**Household income *per capita* (minimum wage)**												
Up to 1	5.6^e^	4.7;6.5	11.9^e^	10.4;13.5	3.0^e^	1.7;4.3	-1.0^c^	-1.8;-0.1	-0.6	-2.1;1.0	0.3	-0.2;0.7
More than 1 to 3	4.2^e^	3.4;5.0	10.4^e^	8.7;12.1	2.7^e^	1.3;4.2	-0.6	-1.6;0.4	-0.6	-2.5;1.3	0.3	0.0;0.6
More than 3	1.4^d^	0.5;2.4	3.2^d^	1.2;5.2	-0.5	-2.5;1.4	-0.3	-1.3;0.7	-0.9	-4.2;2.5	0.3^c^	0.1;0.6
**Place of residence**												
Urban	8.7^f^	7.0;10.3	12.2^f^	9.9;14.6	5.7^f^	3.7;7.8	-0.7	-2.1;0.8	1.9	-0.7;4.4	0.2	-0.4;0.8
Rural	3.8^f^	3.2;4.4	9.6^f^	8.2;11.0	1.7^e^	0.6;2.8	-0.7^d^	-1.4;-0.1	-1.1	-2.4;0.3	0.3^d^	0.0;0.6

a) Only individuals who reported the loss of at least one tooth were considered; b) Absolute difference between the prevalence in 2019 and 2013; c) Indigenous and yellow race/skin color represented 1.46% of the sample and were not shown due to lack of representativeness of such groups in the National Health Survey (PNS); d) P-value < 0.05; and) P-value < 0.01; f) P-value < 0.001; g) 95%CI: 95% Confidence interval.

## Discussion

In general, the 2019 results show better oral health indicators among younger people, those with higher education, higher income and urban residents. There was an improvement in the indicators in the period, with an increase in the prevalence of Brazilians who reported good oral hygiene practices, including brushing their teeth at least twice a day and using a toothbrush, toothpaste and dental floss for oral hygiene, as well as an increase in the prevalence of individuals who self-reported good oral health conditions and a slight reduction in the prevalence of self-reported edentulism.

Regarding the self-report of oral hygiene practices, it is noteworthy that individual preventive care is associated with a lower need for specialized dental treatment.^11^ However, for an adequate oral hygiene, it is necessary to have access to a toothbrush, toothpaste and dental floss.

The incidence of oral diseases and tooth loss is directly linked to the consumption of oral hygiene products. The use of such products, in turn, is significantly associated with age, education and family income,^12^ meaning that sociodemographic aspects directly impact the acquisition of these materials. Thus, it is possible that the increase in such access, observed in the comparison between 2013 and 2019, is the result of public policies for income redistribution, as well as the offer of these products to the population by the Brazilian National Health System, as provided for in the National Oral Health Policy.^2^ ^,^ ^5^

Regarding the self-perception of oral health, most of the population aged ≥ 18 years considered their oral health to be good or very good, with an increase in this prevalence compared to 2013. Different aspects affect this perception, such as the history of problems and dental treatment, tooth loss, pain and age.^13^ The results of this research showed that negative self-assessment increased with age. This fact may be related to a greater self-perception of tooth loss and use of prostheses, and lower self-perception of periodontal diseases and caries or other asymptomatic conditions.^14^ ^,^ ^15^ In this sense, it is argued that there may be a lack of knowledge about the real clinical condition of oral health, which directly influences preventive behavior and care, as well as the interest in accessing dental services.^16^ Therefore, patient-centered health assessment is essential, but it must be considered within the limitations of self-perception and associated factors.^14^

However, it is still clear that the population group with greater difficulties regarding oral health prevention practices, access to hygiene products and reports of good oral health are, in general, the most disadvantaged in Brazilian society in terms of age, education, place of residence and race/skin color. Nico et al.^1^ demonstrated, in a national study with data from the 2013 PNS, worse hygiene practices and worse self-perception of oral health among males, elderly people of Black and Brown race/skin color, with less education and living in rural areas. Bueno et al.,^17^ in a survey with adults in Brazilian capitals using data from the 2010 SB Brasil study, highlighted the significant correlation between social equity and oral health, highlighting the importance of reducing inequities for adequate health promotion. In other words, the prevalence of oral diseases is multifactorial, meaning that contextual, social, environmental and individual aspects must be considered.^18^ For example, such aspects can be verified when one observes that the high prevalence of dental caries occurs among groups who are poorer, less educated, female and of Black and Brown race/skin color.^19^

Advances are observed in the comparative results of the PNS. When evaluating education, there was an increase in brushing in all strata, especially those with lower levels of education. Considering that education is an important component of the social determinants of health, public policies can be seen to address health inequities^20^ and, consequently, influence cultural and behavioral changes related to preventive oral health care.

There was a slight reduction in the percentages of edentulism in relation to the 2013 PNS, with higher prevalence among women, the elderly, people with no schooling or with incomplete elementary education, with a household income *per capita* of up to 1 MW and living in rural areas. Despite the methodological differences, the results of national epidemiological surveys on oral health, carried out in 2003 and 2010, are similar to the present study. In 2003, the number of teeth lost in the adult population was higher among the elderly, women, rural residents, the poorer and less educated.^6^

When the results of the 2010 SB Brasil are analyzed, an improvement in the oral health conditions of Brazilians is observed. However, the prevalence of tooth loss among women, the elderly, people with lower income and low education remained high.^21^ An analysis of the results suggests that the oral health conditions of an important portion of the population still shows little change. The high prevalence of edentulism in the elderly may reflect little or no exposure to preventive measures in the past, such as fluoridation of water supplies.^22^ Additionally, low education, socioeconomic disadvantages and demographic characteristics can play an important role in people's lives, influencing the evolution of oral diseases and culminating in tooth loss.^6^ ^,^ ^22^

Tooth loss can negatively impact the quality of life, generating difficulties in phonation, chewing, and may lead to a decrease in self-esteem and social exclusion.^23^ Despite the improvement in the oral health conditions of the Brazilian population, edentulism is still a problem of great importance in public health.

As a consequence of tooth loss, the need for prosthetic treatments in health services presents a challenge for managers regarding the offer of oral health care focused on the needs of the population, mainly due to the great demand.^24^ In addition, the increased need for dental prosthesis is directly related to the increase in treatment costs.^24^

There were no significant differences in the proportion of people who reported using a dental prosthesis compared to that observed in 2013. On the other hand, the proportion of individuals who reported intense or very intense difficulty in eating showed an increase of 0.3 percentage point in relation to 2013. In general, tooth loss, use of a dental prosthesis and difficulty in chewing are events that are, not rarely, identified concomitantly in people's oral health history. Sheiham et al.^25^ emphasize that the use of a dental prosthesis can directly influence chewing ability, texture perception and food taste. In the elderly population, tooth loss is an outcome resulting from exposure to caries, periodontal disease, periapical disease or trauma, which lead to chewing problems, worse self-perception of health, need for prosthetic rehabilitation and lower satisfaction with appearance. Therefore, it should not be accepted as a normal consequence of the aging process.^26^ ^,^ ^27^

A qualitative study with 66 adults and elderly people in Porto Alegre,^28^ published in 2019, found that among adults with partial tooth loss and elderly people without prosthetic rehabilitation or with partial rehabilitation, tooth loss was perceived as a problem for life. Tooth loss implied limitations in chewing, physical appearance, speech and smiling, social interaction and employment, in addition to embarrassment and pain. For the authors, this condition affected people’s interaction with the world and their daily activities. In this sense, the authors also cite an apparent resignation of the elderly in relation to tooth loss and acceptance of inadequate prosthesis, even with possible discomfort.

Studies in Brazil, carried out in 2002, 2003 and 2010, and specifically in the state of São Paulo, in 2005, pointed to a higher prevalence of tooth loss in females, as well as a worse perception of chewing among female individuals, of Black race/skin color and with a low level of education.^6^ ^,^ ^29^ It is argued that women take up a prominent role in personal and intrafamily care, showing greater concern with the appearance and health of teeth and mouth. As a result, women tend to seek health services more often, a fact that can expose the existence of morbidities in the oral cavity, overtreatment and an increase in tooth extractions.^6^ ^,^ ^21^

This study used data from large national surveys, which did not allow for clinical examinations, which may characterize a limitation of the study. It is also important to highlight that people's behavior is influenced by subjective elements, and these elements can evidently interfere with personal experiences about oral health, which may lead the respondents to overestimate their adherence to socially desirable behaviors and practices. In spite of that, the study provides a recent overview of the self-reported oral health in a representative national sample, and the validity of self-perception in oral health is recognized in the literature.^15^ ^,^ ^30^

This study found that there are still important inequalities in health indicators in the population, with better indicators among the younger, more educated, with higher income and urban area residents. The 2019 results show an improvement compared to 2013, especially with regard to oral hygiene practices and access to products. However, it became clear that a portion of the population is still excluded from the advances achieved. Low education and income, older age, race/skin color, and gender differences proved to be the basis for the maintenance of disparities in oral health, reinforcing the need to strengthen health promotion public policies and equity in access to oral health services.

## References

[1] Nico LS, Andrade SSC de A, Malta DC, Pucca GA, Peres MA (2016). Saúde Bucal autorreferida da população adulta brasileira:
resultados da Pesquisa Nacional de Saúde 2013. Cien Saude Colet.

[2] Bordin D, Fadel CB (2012). Pacto pela saúde no Brasil: uma análise descritiva da progressão
dos indicadores de saúde bucal. Rev Odontol UNESP.

[3] Andrade de FR, Narvai PC (2013). Inquéritos populacionais como instrumentos de gestão e os modelos
de atenção à saúde. Rev Saude Publica.

[4] Nascimento S do, Frazão P, Bousquat A, Antunes JLF (2013). Condições dentárias entre adultos brasileiros de 1986 a
2010. Rev Saude Publica.

[5] Chaves SCL, Almeida AMF de L, Rossi TRA, Santana SF de, Barros SG de, Santos CML (2017). Política de Saúde Bucal no Brasil 2003-2014: cenário, propostas,
ações e resultados. Cien Saude Colet.

[6] Barbato PR, Nagano HCM, Zanchet FN, Boing AF, Peres MA (2007). Perdas dentárias e fatores sociais, demográficos e de serviços
associados em adultos brasileiros: uma análise dos dados do Estudo
Epidemiológico Nacional (Projeto SB Brasil 2002-2003). Cad Saude Publica.

[7] Damacena GN, Szwarcwald CL, Malta DC, Souza PRB de, Vieira MLFP, Pereira CA (2015). O processo de desenvolvimento da Pesquisa Nacional de Saúde no
Brasil, 2013. Epidemiol Serv Saúde.

[8] Souza-Júnior de PRB, Freitas MPS de, Antonaci G de A, Szwarcwald CL (2015). Desenho da amostra da Pesquisa Nacional de Saúde
2013. Epidemiol Serv Saude.

[9] Brasil, Instituto Brasileiro de Geografia e Estatística -
IBGE (2020). Pesquisa nacional de saúde 2019: percepção do estado de saúde, estilos
de vida, doenças crônicas e saúde bucal. Brasil e grandes regiões.

[10] Stopa SR, Szwarcwald CL, Oliveira de MM, Gouvea E de CDP, Vieira MLFP, Freitas MPS de (2020). Pesquisa Nacional de Saúde 2019: histórico, métodos e
perspectivas. Epidemiol Serv Saude.

[11] Azevedo MB de, Pinto R da S, Abreu MHNG de, Lucas SD (2020). Factors associated with the needs of specialised dental treatment
among adults aged 35-44 years old in the state of Minas Gerais, Brazil: a
multilevel cross-sectional study. Cien Saude Colet.

[12] Souza LMM de, Nóbrega LM da, Barbosa KGN, Carneiro FG, Bento PM, DAvila S (2014). Avaliação do consumo e custo de produtos de higiene bucal para
população de um município no Nordeste brasileiro. Arqu Odontol.

[13] Locker D, Maggirias J, Wexler E (2009). What frames of reference underlie self-ratings of oral
health. J Public Health Dent.

[14] Schützhold S, Holtfreter B, Schiffner U, Hoffmann T, Kocher T, Micheelis W (2014). Clinical factors and self-perceived oral health. Eur J Oral Sci.

[15] Haikal DS, Paula de AMB, Martins AME de BL, Moreira AN, Ferreira EF e (2011). Autopercepção da saúde bucal e impacto na qualidade de vida do
idoso: uma abordagem quanti-qualitativa. Cien Saude Coletiva.

[16] Vered Y, Sgan-Cohen HD (2003). Self - perceived and clinically diagnosed dental and periodontal
health status among young adults and their implications for epidemiological
surveys. BMC Oral Health.

[17] Bueno RE, Moysés ST, Bueno PAR, Moysés SJ (2014). Determinantes sociais e saúde bucal de adultos nas capitais do
Brasil. Rev Panam Salud Publica.

[18] Singh A, Peres MA, Watt RG (2019). The Relationship between Income and Oral Health: A Critical
Review. J Dent Res.

[19] Boing AF, Bastos JL, Peres KG, Antunes JLF, Peres MA (2014). Determinantes sociais da saúde e cárie dentária no Brasil:
revisão sistemática da literatura no período de 1999 a 2010. Rev Bras Epidemiol.

[20] Barreto ML (2017). Desigualdades em Saúde: uma perspectiva global. Cien Saude Colet.

[21] Peres MA, Barbato PR, Reis SCGB, Freitas CHS de M, Antunes JLF (2013). Perdas dentárias no Brasil: análise da Pesquisa Nacional de Saúde
Bucal 2010. Rev Saude Publica.

[22] Peres MA, Antunes JLF, Peres KG (2006). Is water fluoridation effective in reducing inequalities in
dental caries distribution in developing countries? Recent findings from
Brazil. Soz Praventivmed.

[23] Gerritsen AE, Allen PF, Witter DJ, Bronkhorst EM, Creugers NHJ (2010). Tooth loss and oral health-related quality of life: a systematic
review and meta-analysis. Health Qual Life Outcomes.

[24] Borges CM, Campos ACV, Vargas AMD, Ferreira EF e (2014). Adult tooth loss profile in accordance with social capital and
demographic and socioeconomic characteristics. Cien Saude Colet.

[25] Sheiham A, Steele JG, Marcenes W, Lowe C, Finch S, Bates CJ (2001). The relationship among dental status, nutrient intake, and
nutritional status in older people. J Dent Res.

[26] Moreira R da S, Nico LS, Tomita NE (2009). Oral health conditions among the elderly in Southeastern São
Paulo State. J Appl Oral Sci.

[27] Silva da DD, Sousa M da LR de, Wada RS (2005). Autopercepção e condições de saúde bucal em uma população de
idosos. Cad Saude Publica.

[28] Bitencourt FV, Corrêa HW, Toassi RFC (2019). Experiências de perda dentária em usuários adultos e idosos da
Atenção Primária à Saúde. Cien Saude Colet.

[29] Braga APG, Barreto SM, Martins AME de BL (2012). Autopercepção da mastigação e fatores associados em adultos
brasileiros. Cad Saude Publica.

[30] Pedro REL, Bos AJG, Padilha DMP, Silva-Filho da IG (2011). Validação de entrevista por telefone para avaliação da saúde
bucal em idosos. RBCEH.

